# Ribosomal RNA processing impairments in a B cell immunodeficient patient with WDR75 variants

**DOI:** 10.70962/jhi.20250061

**Published:** 2026-05-06

**Authors:** Nidia Moreno-Corona, Alice Valagussa, Lucie Poggi, Orestes Lopez-Ortega, Cécile Masson, Mohammed Zarhrate, Alexis Bertrand, Capucine Picard, Patrick Revy, Damien Bodet, Jérémie Rouger, Martin Castelle, Alain Fischer, Marie-Françoise O'Donohue, Sven Kracker

**Affiliations:** 1 Laboratory of Human Lymphohematopoiesis, INSERM UMR 1163, Imagine Institute, Université Paris Cité, Paris, France; 2 Laboratory of Lymphocyte Activation and Susceptibility to EBV Infection, INSERM UMR 1163, Imagine Institute, Université Paris Cité, Paris, France; 3 INSERM UMR-S1151, CNRS UMR-S8253, Institut Necker Enfants Malades, Université Paris Cité, Paris, France; 4 Departamento de Biología Celular, Cinvestav-IPN, San Pedro Zacatenco, Mexico City, Mexico; 5 Bioinformatics Core Facility, Institut Imagine-Structure Fédérative de Recherche Necker, INSERM U1163 et INSERM US24/CNRS UAR3633, Université Paris Cité, Paris, France; 6 Genomics Core Facility, Institut Imagine-Structure Fédérative de Recherche Necker, INSERM U1163 et INSERM US24/CNRS UAR3633, Université Paris Cité, Paris, France; 7 Laboratory of Genome Dynamics in the Immune System, Equipe Labellisée Ligue Contre le Cancer, INSERM UMR 1163, Imagine Institute, Université Paris Cité, Paris, France; 8 French National Reference Center for Primary Immune Deficiencies, Necker-Enfants Malades University Hospital, Assistance Publique-Hôpitaux de Paris, Paris, France; 9 Study Center for Primary Immunodeficiencies, Hôpital Necker-Enfants Malades, Assistance Publique-Hôpitaux de Paris, Paris, France; 10 Department of Pediatric Immuno-Haematology and Rheumatology, Reference Center for Rheumatic, AutoImmune and Systemic Diseases in Children, Hôpital Necker-Enfants Malades, Assistance Publique-Hôpitaux de Paris, Paris, France; 11 CHU de Caen Normandie, Onco-Immunohématologie Pédiatrique, Caen, France; 12 Imagine Institute, INSERM UMR 1163, Université Paris Cité, Paris, France; 13 Collège de France, Paris, France; 14 MCD, Centre de Biologie Intégrative, University de Toulouse, CNRS, Toulouse, France

## Abstract

Ribosomopathies are rare genetic disorders associated with abnormalities in the production and function of ribosomes. We describe here our study of a patient presenting with hypogammaglobulinemia and autism spectrum disorder. Genetic analyses revealed compound-heterozygous variants in the *WDR75* gene. *WDR75* encodes a component of the SSU processome, a large ribonucleoprotein complex involved in the early stages of small ribosomal subunit assembly. Functional studies in cells derived from the patient and a cell line modified by CRISPR/Cas9 showed altered processing of pre-rRNA (A0 cleavage) and increased expression of p21, suggesting a predisposition to nucleolar stress and partial activation of the p53 pathway. While these observations do not prove causality between genotype and phenotype, they suggest a possible association between *WDR75* genetic variants and nuclear stress, which may contribute to the clinical presentation.

## Teaser

Moreno-Corona et al. identify compound-heterozygous WDR75 variants in a patient with hypogammaglobulinemia and autism spectrum disorder. These variants impair pre-ribosomal RNA processing and trigger nucleolar stress, suggesting a new ribosomopathy.

## Introduction

The dynamic regulation of ribosome levels is critical for protein synthesis in human cells, supporting cell growth and proliferation and playing an essential role during development. Human ribosomes are composed of four ribosomal RNAs (rRNAs) and 79 ribosomal proteins (RPs), which assemble into the small (40S) and large (60S) subunits. Defects in the transcription and processing (modification) of pre-rRNA and ribosome assembly are the cause of ribosomopathies (1). The biogenesis of human ribosomes starts in the nucleolus by the synthesis of a primary transcript (the 47S pre-rRNA) by RNA polymerase I. This precursor contains the sequences for 18S, 5.8S, and 28S rRNAs, flanked by external (5′ETS, 3′ETS) and internal (ITS1, ITS2) transcribed spacers, which are sequentially removed during processing. The 5′ETS plays a crucial role in early ribosome assembly by recruiting assembly factors and serving as a dynamic structural scaffold for the formation of early small subunit (SSU) intermediates (2). Cryo-electron microscopy (cryo-EM) studies in eukaryotes have revealed a conserved structural organization of the SSU processome (3). This dynamic platform is composed of the U3 snoRNA assembled to several U3-ribonucleoprotein complexes (UTPA, UTPB, UTPC), comprising >70 assembly factors in human cells. The SSU processome allows both the binding of the first RPs of the small ribosomal subunits to the 18S rRNA sequence and the removal of the 5′ETS through a series of endo- and exonucleolytic processing steps (4). The U3 snoRNP-associated WD repeat protein (Nan1/Utp17) is the yeast ortholog of human WDR75 and is required for 18S rRNA biogenesis (5). WDR75 is part of the UTPA complex, which serves as a foundation to initiate the assembly of the SSU processome on the 5′ETS and is also required for optimal 47S pre-rRNA transcription in human cells (6). Cryo-EM structures of human SSU processome have shown that downstream of the cotranscriptional A′ cleavage site, an evolutionarily conserved motif within the 5′ETS is specifically recognized by a composite binding site formed by UTP4 and WDR75 (3, 7). Errors in ribosome biogenesis can cause nucleolar stress, altering nucleolar morphology and stabilizing p53 (8). It has been proposed that WDR75 plays a role in the cellular response to nucleolar stress (6).

In this study, we identified compound-heterozygous variants in the *WDR75* gene in a patient with a syndromic immune deficiency. We investigated their impact on ribosomal biogenesis and cellular processes related to WDR75 function.

## Results

### Clinical case

**Figure 1.**
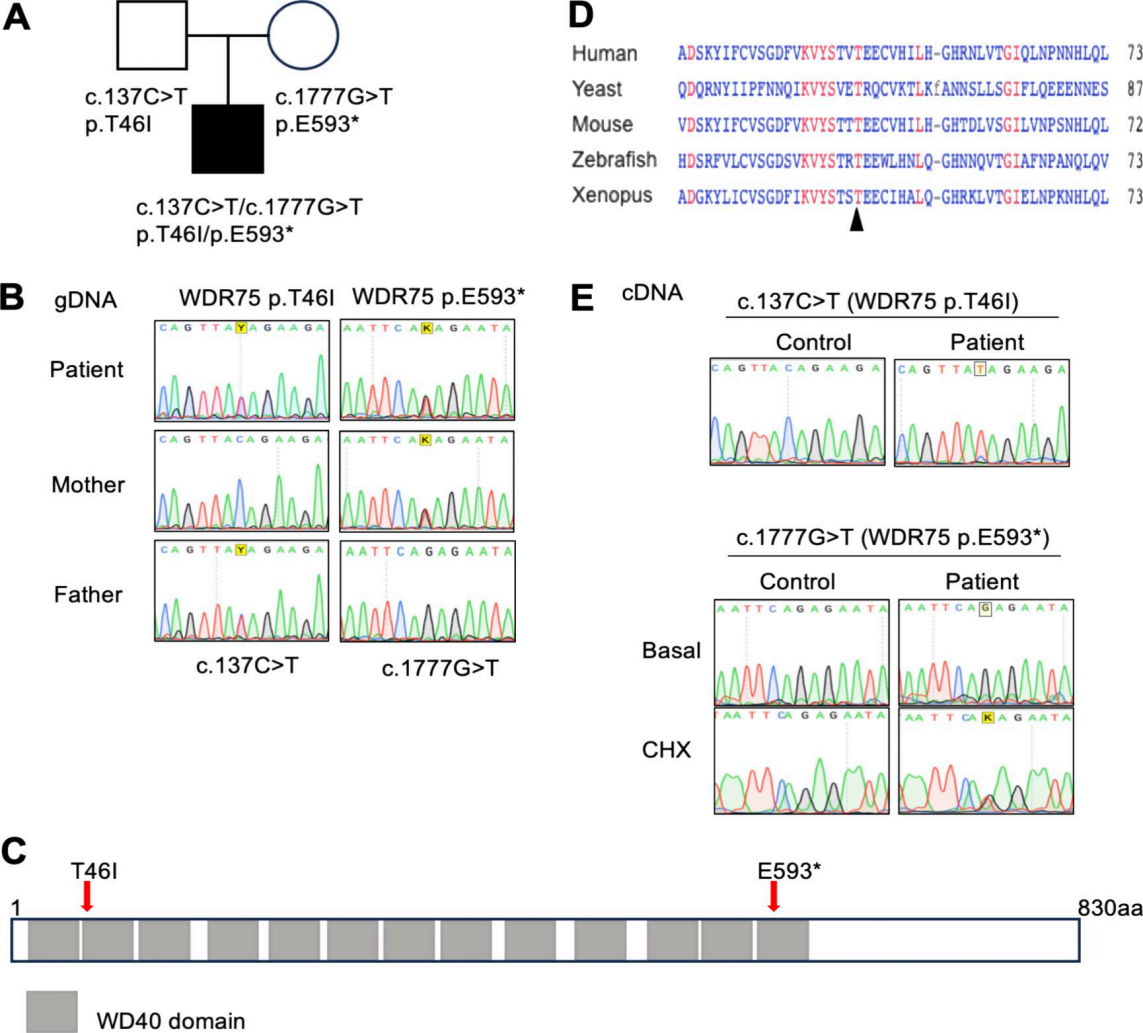
**Identification of compound-heterozygous variants in the *WDR75* gene in an immunodeficient patient with syndromic features. (A–E)** Pedigree indicating carrier status of *WDR75* variants (A). Sanger sequencing from gDNA (B). Schematic presentation of the WDR75 protein indicating the location of WDR75 variants and WD40 repeats (C). Alignment of WDR75 with orthologs; arrowhead indicates the location of the human WDR75 p.T46I variant (D). Sanger sequencing of RT-PCR products with/without CHX treatment (100 µg/ml for 2 h) (E).

**Figure S1.**
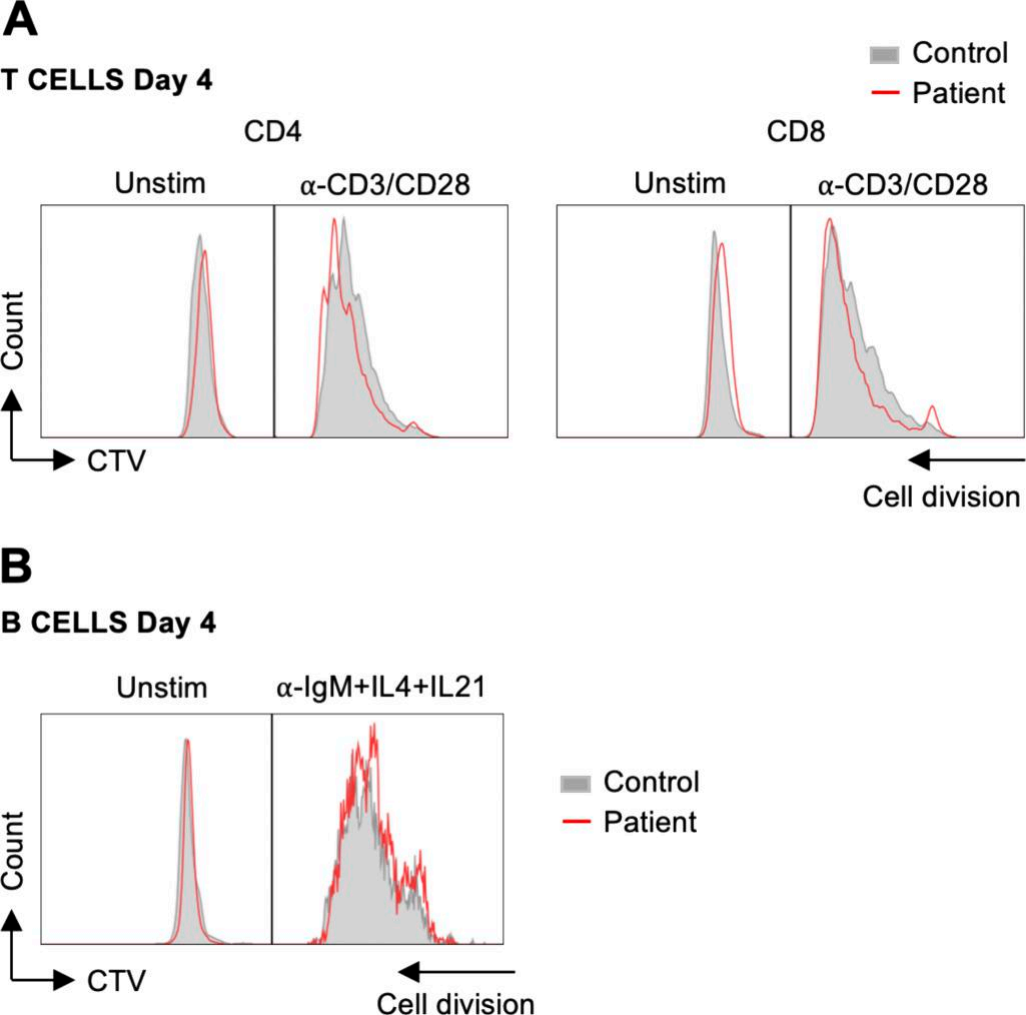
**B cell and T cell proliferation are unaffected in the patient. (A)** PBMCs from the patient and healthy control were labeled with CTV and stimulated with anti-CD3/CD28 beads for 4 days. Proliferation history profiles of gated CD4^+^ and CD8^+^ T cells are shown (*n* = 2). **(B)** PBMCs from the patient and a healthy donor were labeled with CTV and stimulated with anti-IgM+IL-4+IL-21. Proliferation history profiles of gated CD19^+^ B cells are shown (*n* = 1).

A male child of unrelated parents presented with recurrent ear, nose, throat, and bronchopulmonary infections since early childhood (2 years of age) (Fig. 1 A). The patient, who has been receiving suboptimal IgG replacement therapy since the age of 7 years, presented at that age with low serum IgG levels (5.8 g/l), while IgA (0.89 g/l) and IgM (0.52 g/l) levels were within normal ranges (Table 1). Prior to the initiation of IgG replacement therapy, deficiencies in IgG1 and IgG3 subclasses were observed and reported (exact values not available). Clinical immunophenotyping at 12 and 14 years of age revealed a low proportion of switched memory B cells (Table 1). Both normal somatic hypermutation frequency and nucleotide substitution pattern were observed in the VH3-23 region of IgM analyzed in CD19^+^CD27^+^ B cells from the patient (Table 2). The proportion of naive B cells was increased. Likewise, a slightly higher proportion of naive CD8 T cells were observed (Table 1). The counts of hemoglobin, leukocytes, monocytes, neutrophils, and platelets in the blood were normal (Table 1). The patient’s T and B lymphocytes proliferated normally upon activation (Fig. S1). At the age of 15, the patient contracted a human herpesvirus 6 infection (log 2.1). In addition to these immunological characteristics, the patient presented with an autism spectrum disorder.

**Table 1.** Hematological and immunological features of the patient

​	​	Patient		Reference values
Age at evaluation (years)	7	12	14	​
**Serum Ig levels**				
IgG* (g/l)	**5.8**	**6.24**	11.43	6.98–15.6
IgA (g/l)	0.89	1.35	1.46	0.53–2.9
IgM (g/l)	0.52	0.67	0.72	0.31–1.62
**Blood parameters**				
Hemoglobin (×10^9^/liter)	​	14.2	14.9	13–17
MCV (fl)	​	NA	88.4	78–96
Leukocytes (×10^9^/liter)	​	7.15	7.58	4.5–13
Platelets (×10^9^/liter)	​	315	291	150–450
Monocytes (×10^9^/liter)	​	NA	0.75	0.3–1
Neutrophils (×10^9^/liter)	​	3.97	4.28	2–8
*T lymphocytes*				
CD3^+^(/μl)	​	**2,534**	1,729	1,000–2,200
CD3^+^CD4^+^(/μl)	​	**1,404**	922	530–1,300
Naive CD4^+^/CD45RA^+^ (%)	​	**71**	66.4	58–70
Memory CD4^+^/CD45RO^+^ (%)	​	**29**	**33.6**	34–67
CD4^+^/CD31^+^CD45RA^+^ (%)	​	49	49.6	43–55
TH1 CD4^+^CD45RA^−^/CXCR3^+^CXCR5^−^ (%)	​	NA	61	35.3–61
TH2 CD4^+^CD54RA^−^/CCR4^+^CCR6^−^ (%)	​	NA	7.5	4.4–13
TH17 CD4^+^CD45RA^−^/CCR4^+^CCR6^+^ (%)	​	NA	9.8	5.5–16.5
Follicular helper CD4^+^CD45RA^−^/CXCR5^+^ (%)	​	NA	**15.6**	16.3–30.2
Treg CD4^+^/CD25^+^CD127^LOW^ (%)	​	NA	9.5	4.2–10.5
CD8^+^ (/μl)	​	**924**	624	330–920
Naive CD8^+^/CCR7^+^CD45RA^+^ (%)	​	**77**	**82**	40–75
Central memory CD8^+^/CCR7^+^CD45RA^−^ (%)	​	3	**1.4**	2–7
Effector memory CD8^+^/CCR7^−^CD45RA^−^ (%)	​	12	**8.1**	11–20
Terminal effector CD8^+^/CCR7^−^CD45RA^+^ (%)	​	8	8.5	7–32
*NK cells*				
CD16^+^CD56^+^ (/μl)	​	342	149	70–480
*B lymphocytes*				
CD19^+^ (/μl)	​	548	478	193–628
Naive B cells (CD27^−^IgD^+^) (%)	​	**92**	**92**	61.6–87.4
Memory B cells (CD27^+^) (%)	​	**3**	7.6	7–29
Unswitched memory CD27^+^IgD^+^ (%)	​	**2**	5.7	2.6–13.4
Switched memory CD27^+^IgD^−^ (%)	​	**2**	**1.6**	4–21.2
Transitional CD24^++^CD38^++^ (%)	​	**9**	4.9	3.9–7.8
Plasmablast CD24^−^CD38^++^ (%)	​	**0.2**	**0**	0.3–1.7
CD19^+^ CD21^−^CD38^−^ (%)	​	1	**0.8**	0.9–3.3

IgG*, under suboptimal IgG replacement therapy; MCV, mean corpuscular volume; NA, not available. Values above or below reference ranges are marked in bold.

**Table 2.** Mutations in V3-23-Cμ transcript sequences from CD19^+^CD27^+^ purified B cells

​	Clones		Mutations		Ratio	Frequency	
	All	Unmutated^a^	Total	per bp (%)	GC/AT	GC	AT
HD1	8	2	65	2.8	1.5	60	40
HD2	8	0	101	4.3	2.2	69.3	30.6
Patient	9	2	135	5.17	2.2	69	31

a Unmutated ≤ 1 mutation.

### Genetic investigation

**Figure S2.**
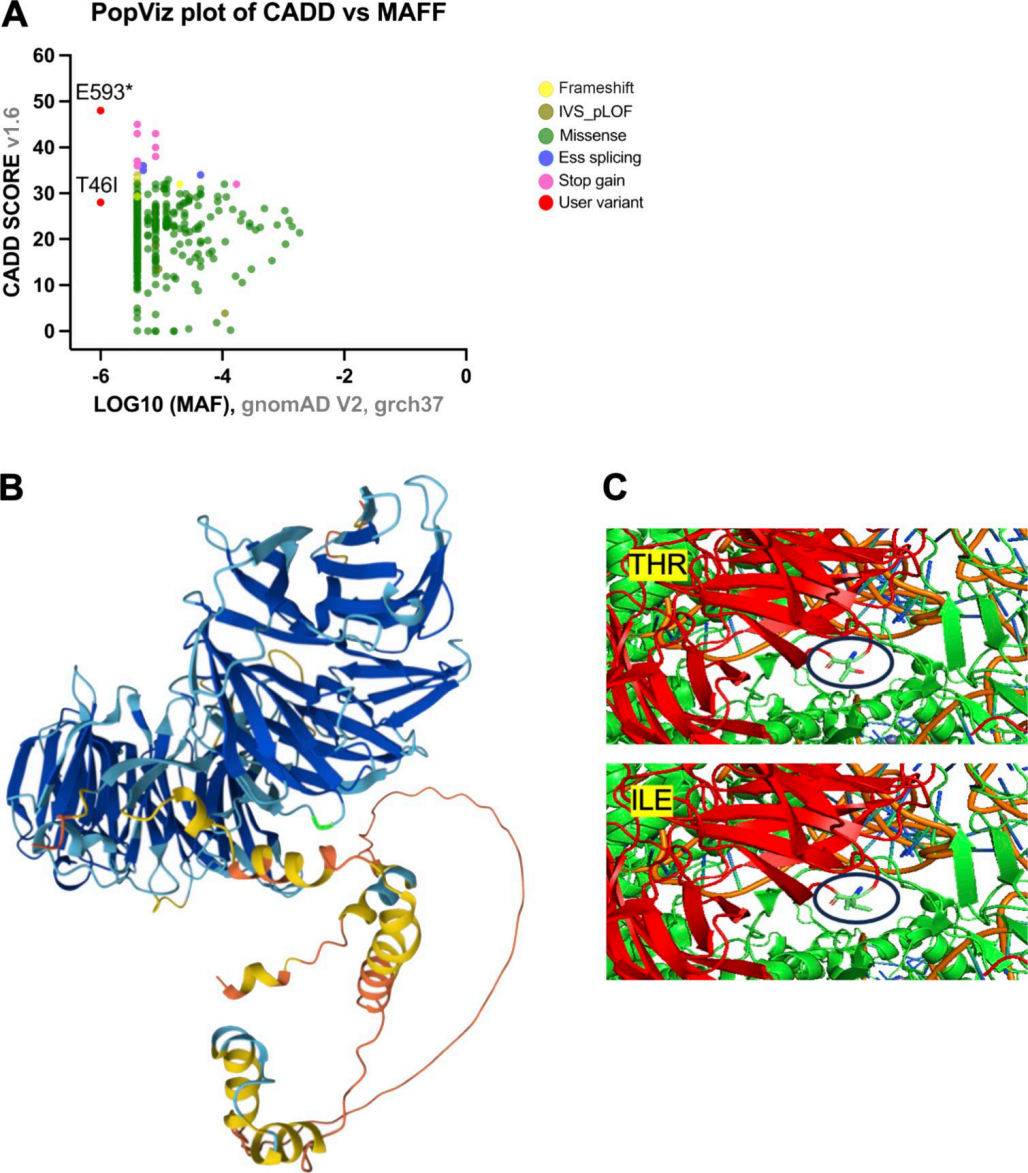
**Evaluation of WDR75 variants based on damage prediction score, allele frequency, and localization. (A)** PopViz plot of WDR75 variants presenting damage prediction score CADD versus MAF using PopViz (http://shiva.rockefeller.edu/PopViz/). **(B)** Location of the WDR75 p.T46I variant highlighted in light green within the predicted AlphaFold protein structure (AF-Q8IWA0-F1-model_v4) (10, 11, 12). **(C)** Location of the WDR75 p.T46I variant within the SSU processome (Q8IWA0). The model was visualized using the PyMOL Molecular Graphics System (version 3.1.0; Schrödinger, LLC). MAF, minor allele frequency.

Whole-exome sequencing of DNA samples from peripheral blood mononuclear cells (PBMCs) of the patient and both parents identified two heterozygous *WDR75* gene variants, a missense variant (Chr2: 190313155; hg19 build 137; NM_032168, exon 2, c. 137C>T, p.T46I), and a nonsense variant (Chr2: 190334123; hg19 build 137; NM_032168, exon 16, c. 1777G>T, p.E593*) in the patient’s DNA. Both variants were confirmed by Sanger sequencing of genomic DNA (Fig. 1, B and C). They were exclusive to the family and not recorded in our internal database (containing 25,429 exomes and 9,136 genomes, November 2025) or in several open-access databases for human genetic variations (Fig. S2 A). No other significant genetic variants were identified by whole-exome sequencing. The WDR75 missense variant p.T46I located within a WD40 repeat–containing domain had a Combined Annotation Dependent Depletion score of 28, PolyPhen-2 score of 0.964, a Sorting Intolerant From Tolerant score of 0.001, and an AlphaMissense score of 0.8144. Structural analyses of WD40 domains suggest that they function as scaffolds for interactions with various proteins, peptides, or nucleic acids (9). Protein sequence alignments of WDR75 orthologs indicated some degree of evolutionary conservation of the WDR75-WD40 domains, particularly at the site of the WDR75 (p.T46I) missense variant (Fig. 1 D). Structural modeling did not reveal significant alterations in the 3D conformation of WDR75 p.T46I or in its known interaction interfaces with pre-rRNA, UTP4, or UTP18 (3, 7, 10, 11, 12) (Fig. S2, B and C). Nonetheless, a potential impact of WDR75 p.T46I on other interaction interfaces cannot be excluded. RT-PCR analysis with mRNA extracted from a patient-derived lymphoblastoid cell line (LCL) confirmed the presence of the WDR75 missense variant (c. 137C>T, p.T46I). However, the nonsense variant of WDR75 (c. 1777G>T, p.E593*) was only detected in mRNA when the LCL was treated with cycloheximide (CHX), suggesting that the mRNA carrying the nonsense variant of WDR75 (c. 1777G>T, p.E593*) is subject to nonsense-mediated decay (Fig. 1 E). Taken together, the potential structural relevance of the p.T46I substitution and the absence of other variants segregating with the disease in genes known to be associated with an inborn error of immunity prompted us to investigate the possible functional effects of the compound-heterozygous WDR75 variants on biological processes.

### Functional impact of WDR75 variants

**Figure S3.**
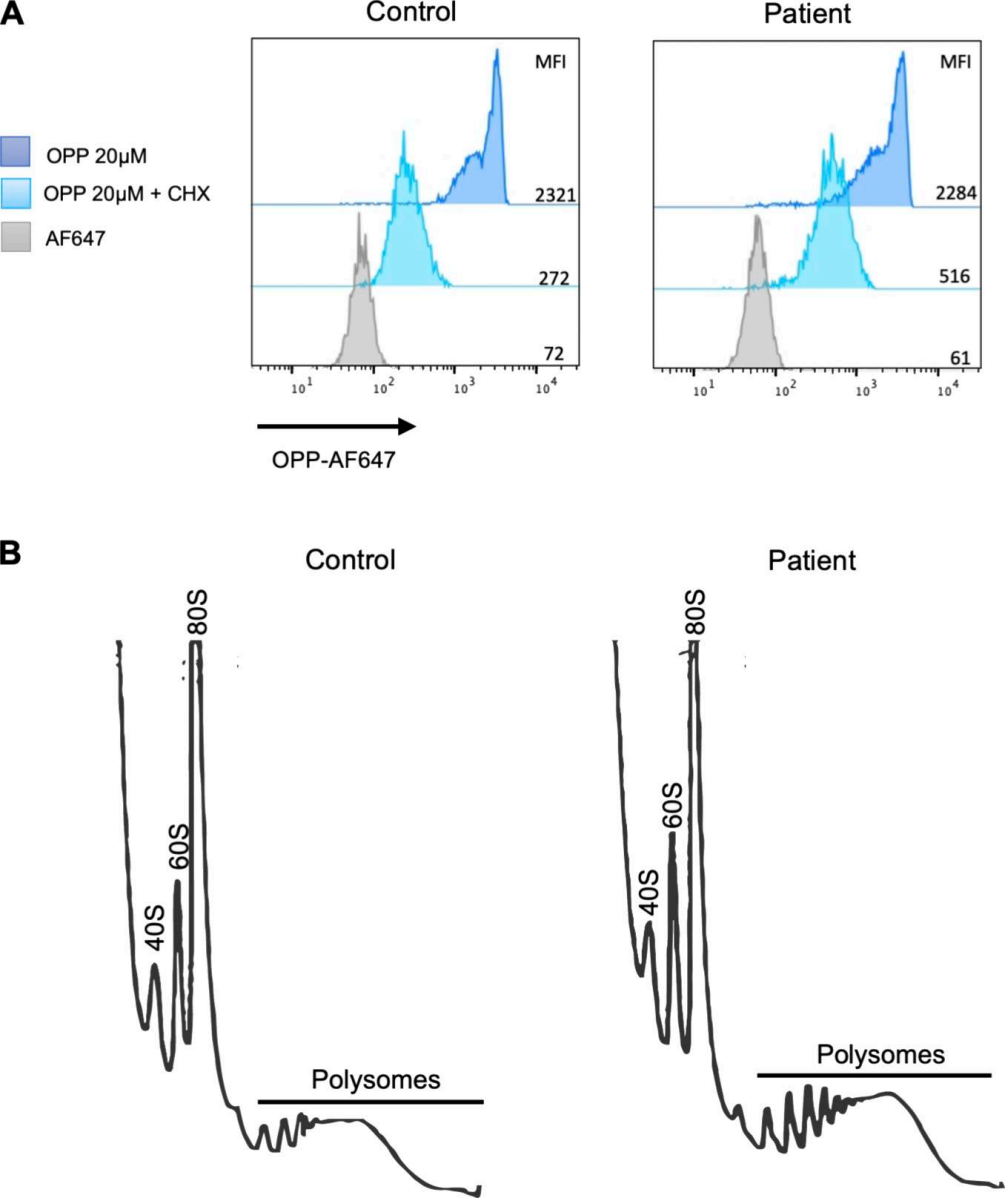
**Patient-derived LCL presents with normal protein synthesis and polysome profile. (A)** LCLs derived from the patient and controls were untreated or treated with CHX 100 µg/ml for 2 h, stained with *OPP* 20 µM for 30 min, and then analyzed by flow cytometry. Representative image of *n* = 3 independent experiments. **(B)** Cytoplasmic extracts from patient- and control-derived LCLs were separated on sucrose gradients in order to analyze their contents in ribosomal subunits and polysome fractions; a representative image of *n* = 2 independent experiments.

**Figure 2.**
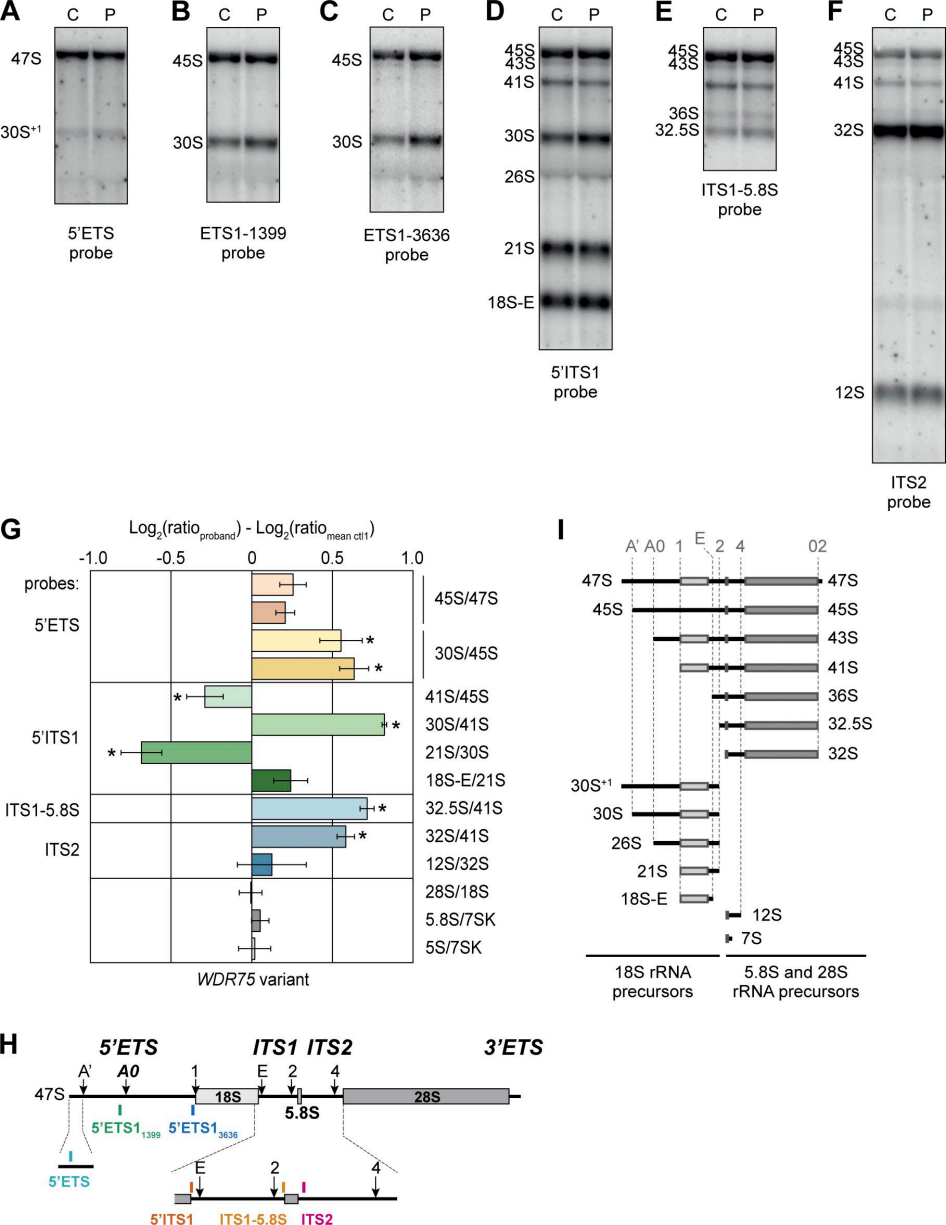
**WDR75 variants affect pre-rRNA processing.** Quantification of pre-rRNA precursors identified by northern blot analysis of LCLs derived from the patient and a control. Indicated radiolabeled probes were used to detect pre-rRNA precursors and mature rRNAs. Statistical analysis was performed using the Mann–Whitney *U* test; *P < 0.05. **(A–C)** 5′ETS probe, 47S transcript; 45S/47S ratio in conjunction with B and C. **(B)** ETS1-1,399 probe, 30S/45S ratio. **(C)** ETS1-3636 probe, 30S/45S ratio. **(D)** 5′ITS1 probe, 41S/45S, 30S/41S, 21S/30S, and 18S-E/21S ratios. **(E)** ITS1-5.8S probe, 32.5S/41S ratio. **(F)** ITS2 probe, 32S/41S and 12S/32S ratios. **(G–I)** Pre-rRNA product-to-precursor ratios obtained for the patient are shown relative to the control. Mean values ± standard error of the mean from *n* = 4 independent experiments, aside from 5S and 5.8S rRNA quantification, for which *n* = 3. Schematic representation of 47S pre-rRNA indicating the sequences for 18S, 5.8S, and 28S rRNAs, flanking 5′ETS and 3′ETS, and ITS1, ITS2 (H). Cleavage sites and probes for northern blots used for A–G are indicated. Schematic representation of 18S, 5.8S, and 28S rRNA precursors (I). Source data are available for this figure: SourceData F2.

Total protein synthesis measured by O-propargyl-puromycin (OPP) labeling and the polysome profile of the patient-derived LCL were normal (Fig. S3). In light of the implication of WDR75 in pre-rRNA processing, we next analyzed pre-rRNA profiles obtained by northern blot from RNA extracted from LCL derived from the patient and an unaffected individual, as described previously (13) (Fig. 2, A–I). The most striking signature obtained for the proband was an accumulation of 30S pre-rRNAs, as seen on the northern blot and attested by an increased 30S/45S ratio (Fig. 2, B–D). As the 5′ETS is processed before the ITS1 in pre-40S particles, accumulation of 30S pre-rRNA in the proband’s cells corresponded to an impaired cleavage of the 5′ETS at site A0, in line with knockdown studies, which have previously implicated WDR75/Utp17 in 5′ETS processing in eukaryotes (14). On the contrary, the first two endonucleolytic cleavages to take place on the primary transcript, which correspond to sites A′ in the 5′ETS and 02 in the 3′ETS, remained unaffected. Maturation of pre-rRNAs of the large ribosomal subunit remained globally unaffected (Fig. 2, E and F), and production of mature 5S, 5.8S, 18S, and 28S rRNAs was similar to that of control cells (Fig. 2 G). Altogether, these data suggest a delayed cleavage at site A0 in compound-heterozygous *WDR75* variant patient cells.

**Figure 3.**
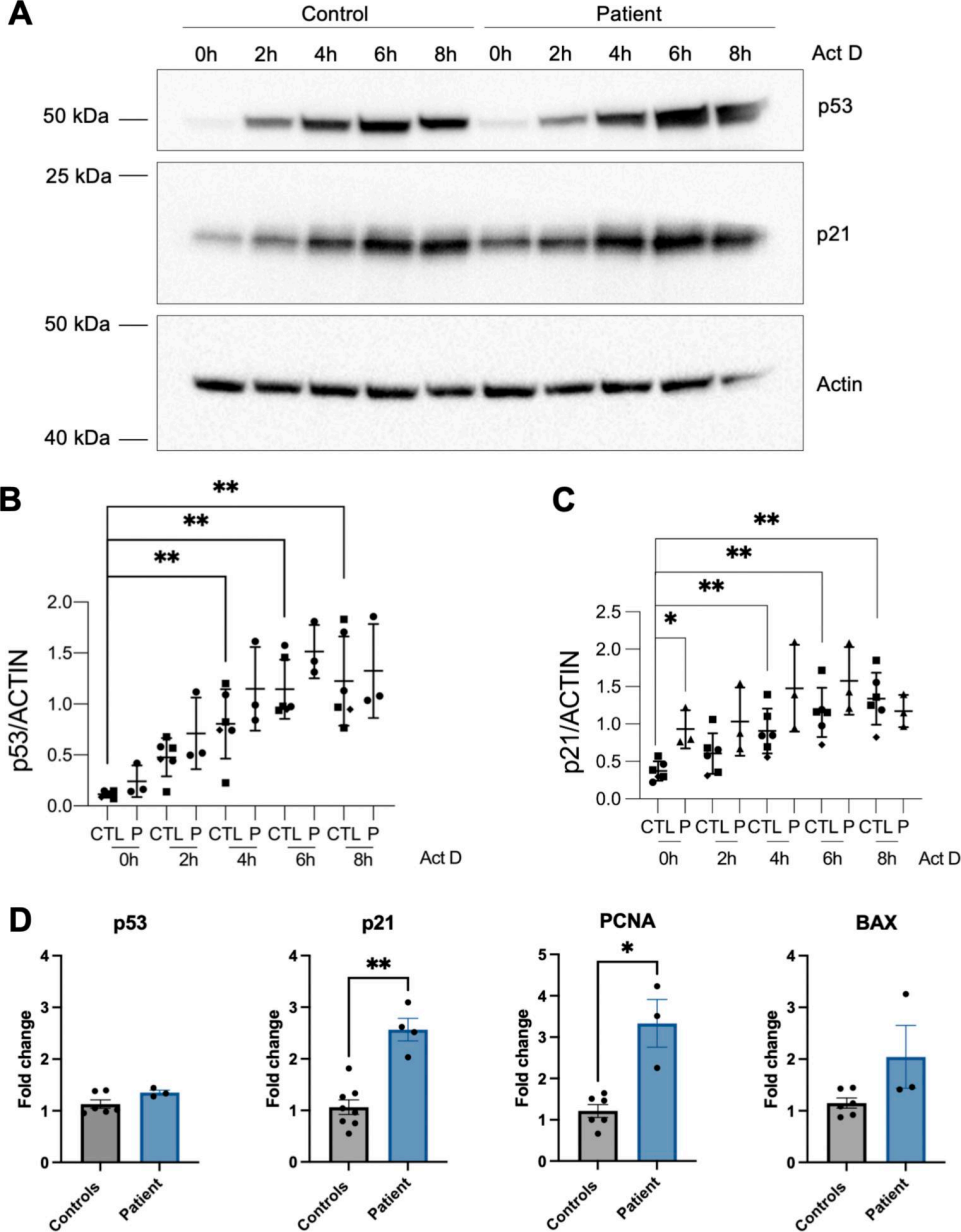
**WDR75 variants are associated with increased p21 expression. (A–D)** Expression of p53 and p21 protein from LCLs from controls and the patient untreated or treated with 5 nM Act D for 2, 4, 6, and 8 h (A). Representative image of *n* = 3 independent experiments (A). Quantification and statistical assessment for all experiments performed in A for p53 (B) and p21 (C) expression. Data were analyzed using the Mann–Whitney *U* test. *P < 0.05; **P < 0.001. Quantitative PCR analysis of *p53*, *p21*, *PCNA*, and *BAX* transcripts from LCLs from controls and the patient (D). *n* = 3 and for p21 *n* = 4 independent experiments ± SD. Data were analyzed using the Mann–Whitney test. *P < 0.05; **P < 0.001. Act D, actinomycin D. Source data are available for this figure: SourceData F3.

WDR75 knockdown has been implicated in activating the p53 pathway (6). As reported, actinomycin D treatment triggered activation of the p53 pathway in LCLs (Fig. 3, A and B). However, the expression of p53 in the patient’s LCL was comparable to that of control cells. A downstream target and effector of p53 is p21, a cyclin-dependent kinase inhibitor. The patient-derived LCL exhibited increased p21 protein expression compared with controls, particularly before actinomycin D treatment (Fig. 3, A and C). Increased *p21* expression was also evident on the mRNA level (Fig. 3 D). Other p53 targets, such as *PCNA* and *BAX*, were also increased at the mRNA level in the patient-derived LCL compared with controls (Fig. 3 D). Taken together, the data suggest increased expression of p53 targets, especially p21, in the patient-derived LCL.

**Figure S4.**
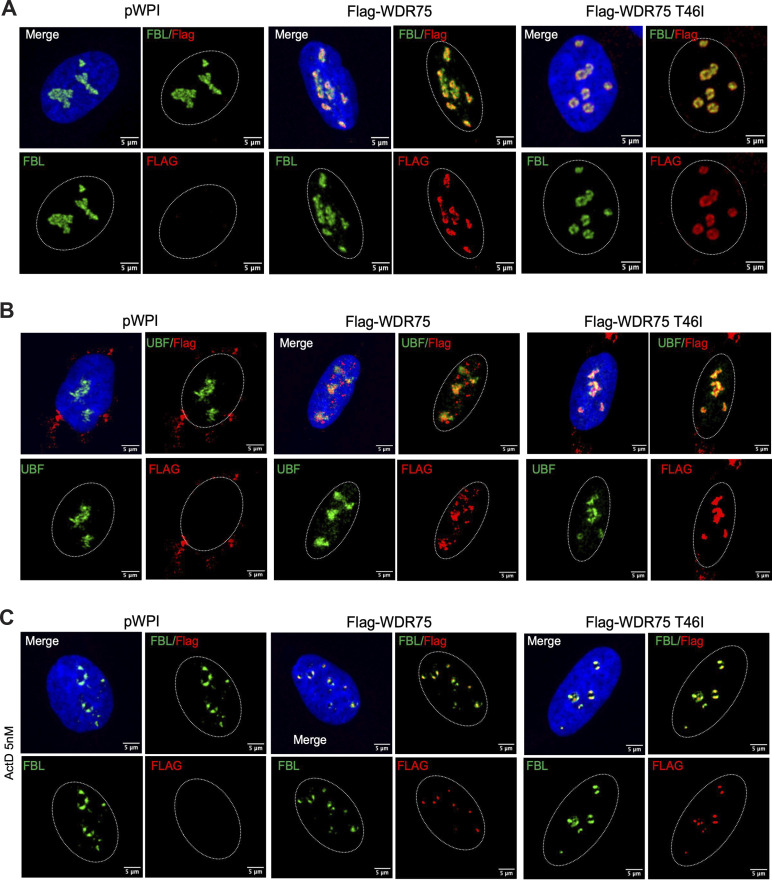
**WDR75 p.T46I variant colocalizes with UBF and fibrillarin. (A)** U2OS cells expressing an empty vector pWPI, Flag-WDR75, or Flag-WDR75 p.T46I were stained for fibrillarin (FBL, green), anti-Flag (red), and nuclei with DAPI (blue); bars, 5 μM. **(B)** U2OS cells expressing an empty vector pWPI, Flag-WDR75, or Flag-WDR75 p.T46I were stained for UBF (green), anti-Flag (red), and nuclei with DAPI (blue); bars, 5 μM. **(C)** U2OS cells expressing a pWPI empty vector, Flag-WDR75, or Flag-WDR75 p.T46I were treated with 5 nM Act D for 4 h and then stained for fibrillarin (FBL, green), anti-Flag (red), and nuclei with DAPI (blue); bars, 5 μM. **(A–C)** Representative images from *n* = 2 independent experiments. Act D, actinomycin D.

Due to the lack of reliable antibodies for detecting endogenous WDR75, studies have used WDR75-tagged proteins to show its nucleolar localization (6, 14). We expressed Flag-tagged WDR75 wild-type (WT) and p.T46I variants in U2OS cells and investigated their nucleolar localization (Fig. S4, A and B). As reported (6, 14), Flag-tagged WDR75 WT proteins were localized at the boundary between the fibrillar center and the dense fibrillar component, where transcription of rRNA by RNA polymerase I occurs (Fig. S4, A and B). Actinomycin D treatment caused both WDR75 WT and p.T46I proteins to redistribute to nucleolar caps (Fig. S4 C). These results suggest that WDR75 p.T46I maintains normal nucleolar localization and responds similar to RNA polymerase I inhibition, although its function in the U3 processing complex might be affected.

**Figure S5.**
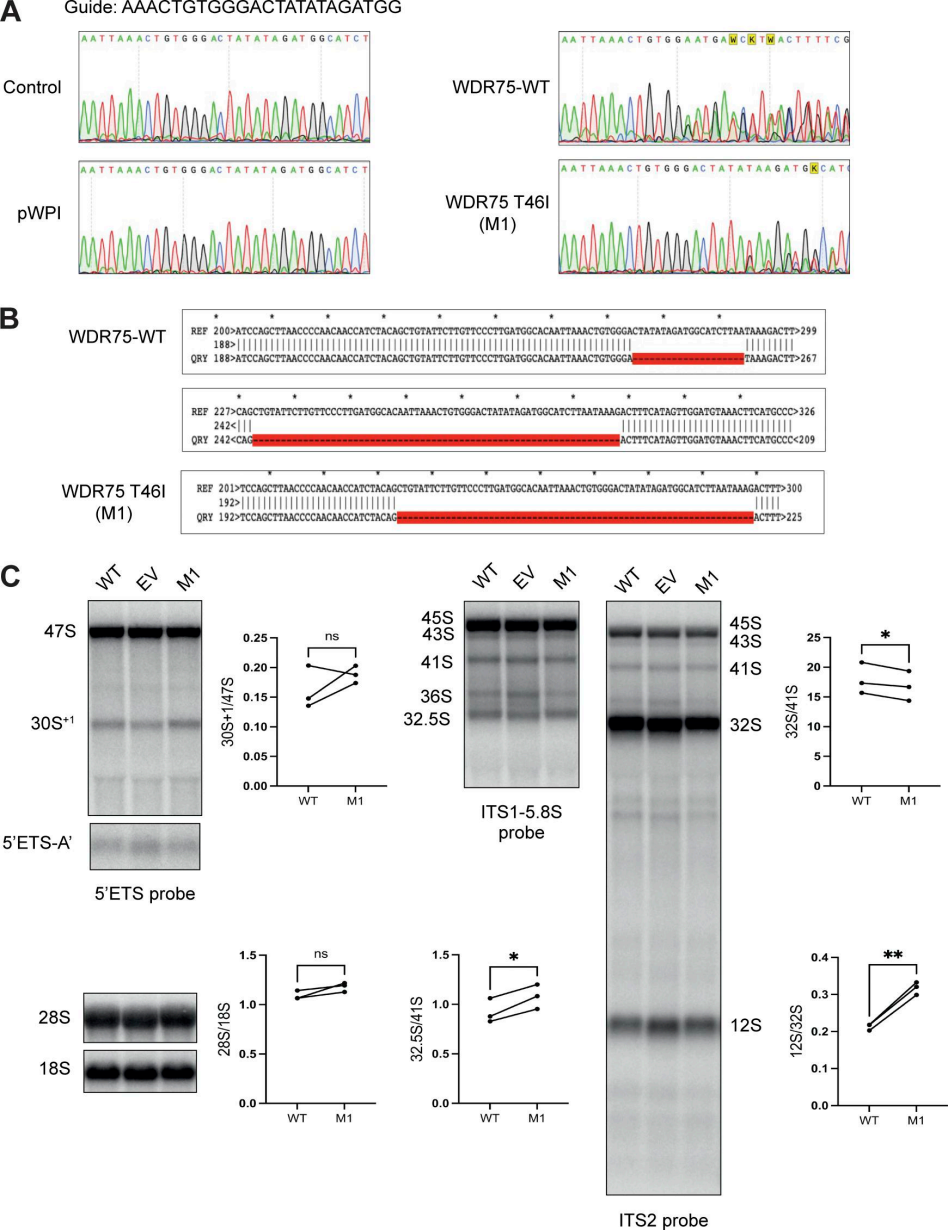
**U2OS cells with inactivation of the *WDR75* gene locus and ectopic expression of WDR75 p.T46I present with altered pre-rRNA processing. (A)** Sanger sequences of gDNA at the site (guide sequence) of CRISPR/Cas9 modification of different U2OS clones ectopically expressing either WDR75 WT or WDR75 p.T46I. **(B)** Alignments of sequencing profiles from TOPO TA cloned RT-PCR products of U2OS cells modified by CRISPR/Cas9. U2OS cells were treated with CHX 100 µg/ml for 2 h before extracting the RNA. **(C)** Northern blot analysis of U2OS cells ectopically expressing a pWPI EV, WDR75 WT, or WDR75 p.T46I (M1). The *WDR75* gene locus was inactivated (see A and B) in WT- and WDR75 p.T46I-expressing U2OS cells. Indicated radiolabeled probes were used to detect pre-rRNA precursors and mature rRNAs. Statistical analysis presented in C was performed using paired Student’s *t* test; *P < 0.05; **P < 0.01. Source data are available for this figure: SourceData FS5.

**Figure 4.**
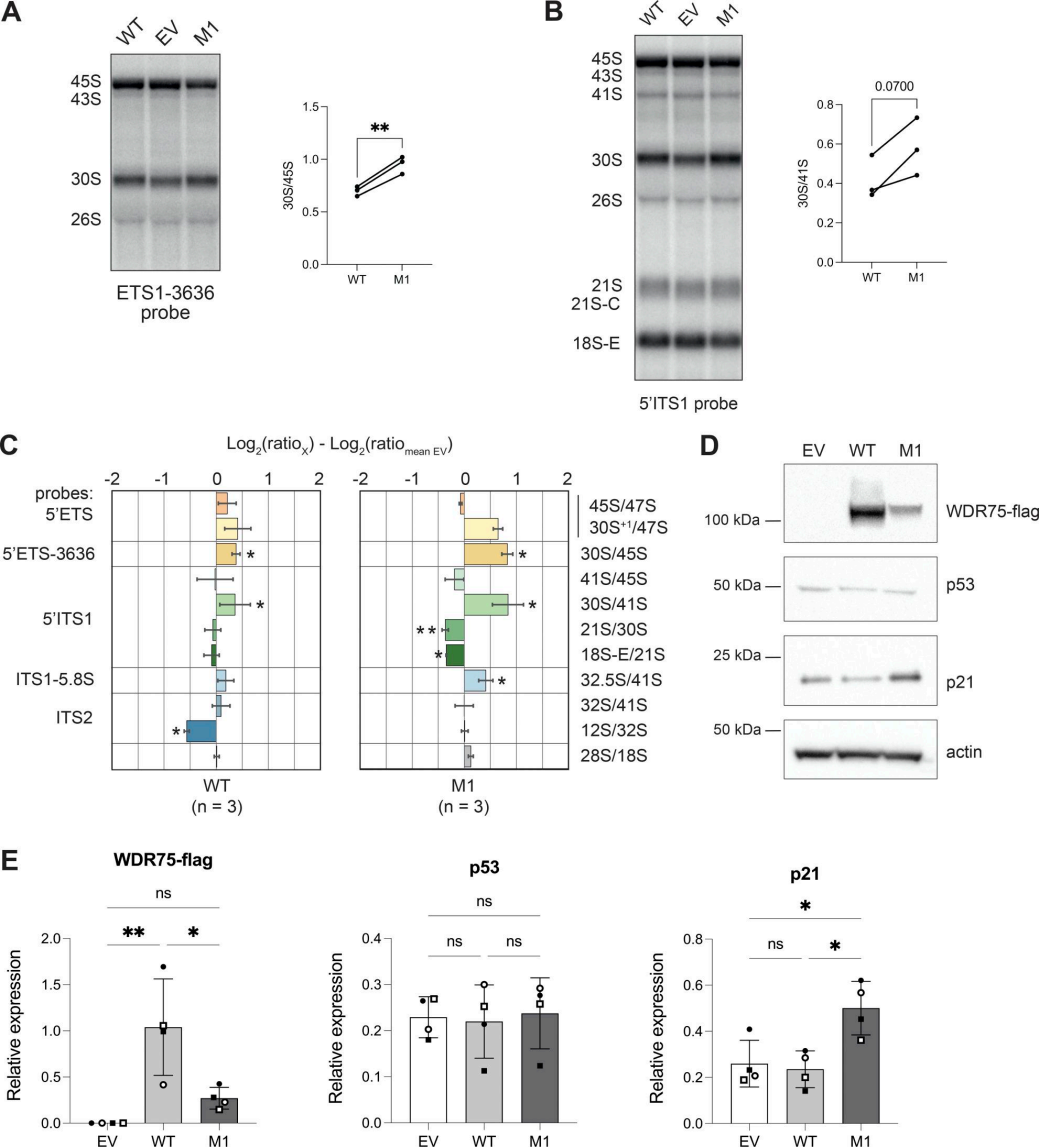
**Altered pre-rRNA processing and increased p21 expression in U2OS cells with *WDR75* inactivation and ectopic expression of WDR75 p.T46I. (A and B)** Northern blot analysis of U2OS cells ectopically expressing a pWPI EV, WDR75 WT, or WDR75 p.T46I. The *WDR75* gene locus was inactivated by CRISPR/Cas9 in WT- and WDR75 p.T46I (M1)-expressing cells. Indicated radiolabeled probes were used to detect pre-rRNA precursors and mature rRNAs. **(A)** ETS1-3636 probe, quantification of the 30S/45S ratio in WT and M1 cells. **(B)** 5′ITS1 probe, quantification of 41S/45S, 30S/41S, 21S/30S, and 18S-E/21S ratios in WT and M1 cells. **(C)** Pre-rRNA product-to-precursor ratios obtained for WT and M1 cells, relative to EV controls. Data represent mean values ± SEM from three independent experiments (*n* = 3). Statistical analysis presented in A–C was performed using paired Student’s *t* test; *P < 0.05; **P < 0.01. **(D)** Western blot analysis for p53, p21, and WDR75-Flag protein expression in the indicated U2OS cells. **(E)** Quantification and statistical analysis of WDR75-Flag, p53, and p21 expression levels from D. Statistical analysis was performed using one-way ANOVA. *P < 0.05; **P < 0.001. EV, empty vector. Source data are available for this figure: SourceData F4.

The observed cellular senescence in WDR75 knockdown cells suggests that the *WDR75* gene is crucial for cellular expansion and vitality (6). Thus, if the missense WDR75 p.T46I variant is associated with the disease, this variant is likely to be hypomorphic. To show that the WDR75 p.T46I protein is associated with reduced functional performance, we targeted the *WDR75* gene locus in U2OS cells ectopically expressing either WDR75 WT or WDR75 p.T46I protein using CRISPR/Cas9. Inactivation of the *WDR75* gene locus was confirmed by Sanger sequencing at the genomic and cDNA level (Fig. S5, A and B). Of note, without ectopic expression of WDR75, we could not generate CRISPR/Cas9-treated U2OS clones with inactivation of the *WDR75* gene locus. In line with the pre-rRNA processing data obtained in the patient LCL, a 30S pre-rRNA accumulation was also observed in a CRISPR/Cas9-treated U2OS clone M1 expressing the missense WDR75 p.T46I variant (Fig. 4, A–C, Fig. S5 C). In addition, increased expression of p21 was observed in this U2OS clone compared with controls, further suggesting a partial loss-of-function of the WDR75 p.T46I variant (Fig. 4, D and E).

## Discussion

In this study, we report a patient harboring compound-heterozygous variants in the *WDR75* gene, presenting with hypogammaglobulinemia and autism spectrum disorder. Functional analyses in patient-derived LCLs revealed an accumulation of 30S pre-rRNA, consistent with impaired cleavage at the 5′ETS A0 site. This observation aligns with previous cryo-EM and knockdown studies implicating *WDR75* in early 5′ETS processing during ribosome biogenesis (3, 6). We also observed elevated levels of p21 in patient LCLs, indicative of p53 pathway activation.

To further validate the functional impact of the identified *WDR75* variants, we generated U2OS cell lines with CRISPR/Cas9-mediated inactivation of endogenous *WDR75*, complemented by ectopic expression of either WT or mutant (T46I) *WDR75*. These engineered cells recapitulated both the pre-rRNA processing defects and p21 accumulation observed in patient-derived cells, providing functional evidence for the pathogenic potential of the identified variants. A partial loss of *WDR75* function may contribute to the patient’s immunological abnormalities and neurodevelopmental phenotype. However, our study does not establish a definitive causal relationship between the patient’s genotype and phenotype consistent with the 2014 Journal of Experimental Medicine guidelines for genetic studies in single patients (15). We cannot exclude the potential influence of additional genetic or environmental factors contributing to the clinical phenotype, despite the absence of other significant variants detected in the whole-exome sequencing analysis.

Several manifestations of ribosomopathies have been attributed to the stabilization of p53, and we hypothesize that elevated p21 levels may impair B and T lymphocyte activation, thereby weakening the immune response. Interestingly, ribosomopathies often present with intellectual disability (1). Autism spectrum disorder, noted in our patient, could share a similar link to ribosomal dysfunction, mainly as other studies have reported rare WDR75 variants in patients with neurodevelopmental delays (16). Several studies in yeast and humans have described a dual role of most UTPA proteins (including WDR75) in both transcription and pre-rRNA processing (14, 17). Along this line, a recent study has highlighted a critical role of ribosome-level regulation during early neurodevelopment (18).

Our patient did not exhibit the hematological abnormalities typically associated with Diamond–Blackfan anemia (DBA; OMIM: 105650) (19, 20, 21). Similarly, other ribosomopathies such as isolated congenital asplenia, characterized by the absence of a spleen at birth without other developmental defects, also lack the hematological features seen in DBA (22), while some mutations in *RPS10* and *RPS23* have been associated in diseases with no hypoplastic anemia (23).

Despite the essential role of ribosomes in all cell types, the tissue-specific manifestations of ribosomopathies remain poorly understood (24). Several nonmutually exclusive mechanisms may contribute to this phenomenon, including differences in gene expression levels and splicing variants across tissues, distinct cellular pathways for sensing defects in ribosome biogenesis, and aberrant or altered translation of specific transcripts (24, 25). Further studies, possibly involving animal models, will be required to establish causality between genotype and phenotype and to clarify any potential tissue-specific effects arising from partial impairment of WDR75 function.

The accumulation of 30S pre-rRNA, indicating defective A0 cleavage in our patient, represents a distinct pre-rRNA processing defect, attesting an impairment at an early step of small ribosomal subunit processing. However, accumulation of the 30S intermediates is not restricted to a loss or a misfunctioning of WDR75, as it could also be due to defects in other assembly factors such as those belonging to the SSU processome (26), or to the knockdown of some RPs whose binding is required during this processing step (27). In DBA, it is the case for mutations in *RPS24*, while mutations of deletions in other RP genes may lead to reduced levels of 18S-E pre-rRNAs (e.g., *RPS10*) or elevated levels of 32S pre-rRNAs (e.g., *RPL5*, *RPL11*), reflecting different disruptions in ribosome biogenesis (21). These findings support the use of pre-rRNA processing profiles as functional biomarkers, valuable not only for distinguishing molecular defects but also for guiding genome-wide approaches in the discovery and characterization of ribosomopathies.

In conclusion, we describe a patient with compound-heterozygous WDR75 variants who exhibits defects in ribosomal biogenesis consistent with a potential ribosomopathy. Additional cases and experimental studies will be required to confirm this association. Our results nonetheless underline the essential role of WDR75 in cellular viability and ribosomal biogenesis.

## Materials and methods

### Blood sample collection and study approval

Peripheral blood samples were collected from the patient and his parents after they provided written, informed consent. Genetic studies and data collection procedures were approved by the local institutional review board (Comité de Protection des Personnes Ile de France II, Paris, France; reference: 2015-01-05; 2015-01-05 MS2) and the French Advisory Committee on Data Processing in Medical Research (Comité Consultatif sur le Traitement de l’Information en matière de Recherche dans le domaine de la Santé, Paris, France; reference: 15.297bis).

### Whole-exome sequencing

Genomic DNA was extracted from PBMCs. Exome libraries were generated with the Twist Bioscience kits (Twist Human RefSeq Exome Kit, 36 Mb) and with the protocol version Twist-NGS Exome-96-12-DOC-001016-Rev1.0-May2018. Briefly, genomic DNA (500 ng) was sheared with an ultrasonicator (Covaris). A total amount of 50 ng of the fragmented and purified double-strand DNA was used to prepare Twist exome libraries as recommended by the manufacturer, but with no initial enzymatic shearing and using adaptors with Unique Dual Identifier (IDT). Barcoded exome libraries were pooled and sequenced with the NovaSeq 6000 system (Illumina), generating paired-end reads (100 bases + 100 bases). After demultiplexing, sequences were aligned to the reference human genome hg19 using the Burrows–Wheeler Aligner. The mean depth of coverage obtained was >132× with >97.9% of the targeted exonic bases covered by at least 15 independent reads and >97.5% covered by at least 30 independent reads. Downstream processing was carried out with the Genome Analysis Toolkit, SAMtools (RRID:SCR_002105), and Picard (RRID:SCR_006525), following documented best practices (https://gatk.broadinstitute.org/hc/en-us/articles/360035894711-About-the-GATK-Best-Practices). All variants were annotated and filtered using PolyWeb, our proprietary annotation software.

### Cell culture

Using SepMate PBMC Isolation Tubes (#85450; STEMCELL Technologies) and Ficoll, we isolated PBMCs from either cytapheresis rings (from a healthy volunteer) or whole blood samples (from the patient). PBMCs were cultured in RPMI-1640 medium (#21875-034; Gibco) supplemented with 10% human serum (type AB) (Sigma-Aldrich), 1× penicillin–streptomycin (#15140-122; Thermo Fisher Scientific).

LCLs were obtained from the Centre de Ressources Biologiques (Necker Campus). LCLs were cultured in RPMI-1640 medium (#21875-034; Gibco) supplemented with 15% fetal bovine serum (FBS, #10270-106; Gibco), 1× penicillin–streptomycin (#15140-122; Thermo Fisher Scientific), and 10 µg/ml gentamicin (#15710-049; Gibco). U2OS cells (RRID:CVCL_0042) were cultured in DMEM with GlutaMAX (#31966-047; Thermo Fisher Scientific), 10% FBS (#10270-106; Gibco), 1× penicillin–streptomycin (#15140-122; Thermo Fisher Scientific), and 10 µg/ml gentamicin (#15710-049; Gibco). All cell lines were tested negative for *Mycoplasma* contamination.

### Lentiviral vectors

Flag-WDR75 WT and Flag-WDR75-T46I constructs were generated by GenScript and inserted in the lentiviral pWPI backbone (RRID:Addgene_12254). The SFR BioSciences Gerland-Lyon Sud (Lyon, France) vector facility produced the lentiviral supernatant, and a multiplicity of infection of 30 was used for further transduction of U2OS cells (RRID:CVCL_0042).

### Extraction of gDNA and mRNA

The gDNA was extracted from the peripheral blood of patients and parents using the FlexiGene DNA kit (51206; Qiagen). The mRNA was extracted using RNeasy Mini Kit (#74904; Qiagen), according to the manufacturer’s instructions. Briefly, 3 million cells from the patient and a healthy individual were plated in a 24-well plate and treated or not with CHX at a concentration of 100 µg/ml (#7698; Sigma-Aldrich). After 2 h of culture, cells were washed once with phosphate-buffered saline (PBS), and mRNA was extracted. The mRNA was processed (including a DNase treatment step), followed by reverse transcription using High-Capacity cDNA Kit (Thermo Fisher Scientific) to generate cDNA according to the manufacturer’s instructions.

### qRT-PCR

Quantitative real-time PCR was performed using the standard protocol. Briefly, a total of 100 ng of cDNA was used to perform a qPCR with Taq Universal SYBR Green Supermix (#1725121; Bio-Rad) and specific primers for each gene: p21 5′-GCA​GAC​CAG​CAT​GAC​AGA​TTT-3′ and 5′-GGA​TTA​GGG​CTT​CCT​CTT​GGA-3′; p53 5′-CCT​CAG​CAT​CTT​ATC​CGA​GTG​G-3′ and 5′-TGG​ATG​GTG​GTA​CAG​TCA​GAG​C-3′; BAX 5′-TCA​GGA​TGC​GTC​CAC​CAA​GAA​G-3′ and 5′-TGT​GTC​CAC​GGC​GGC​AAT​CAT​C-3′; PCNA 5′-CAA​GTA​ATG​TCG​ATA​AAG​AGG​AGG-3′ and 5′-GTG​TCA​CCG​TTG​AAG​AGA​GTG​G-3′. Endogenous human GAPDH 5′-AAG​GTG​AAG​GTC​GGA​GTC​AAC-3′ and 5′-GGG​GTC​ATT​GAT​GGC​AAC​AAT​A-3′ was used as a housekeeping gene. The data were analyzed using the ΔΔCt method and normalized to GAPDH levels.

### PCR and sequencing

Genomic DNA and cDNA were amplified using specific primers for each different mutation: for genomic DNA, WDR75 ex2 5′-ACT​TGA​GGC​CAG​GAG​TTC​AA-3′ and 5′-TGG​GGT​TAA​GCT​GGA​TTC​CA-3′; WDR75 ex16 5′-TCA​GTG​GAG​TGG​AAT​GCA​AA-3′ and 5′-AAG​TGT​TGG​GGC​AAA​GAA​GG-3′; for the cDNA, 5′-CGG​CAG​CGA​GTT​GAA​CTT​TA-3′ and 5′-GGT​TGT​TGG​GGT​TAA​GCT​GG-3′; 5′GCT​GTG​CAT​TGG​AGT​GGA​AT-3′ and 5′-TCA​GGG​ACA​TCT​CGT​GGA​AC-3′. The PCR products were used in the subsequent sequencing BigDye reaction (Terminator v3.1 Cycle Sequencing Kit; Applied Biosystems). The sequence reaction was read using the Applied Biosystems 3500 Series Genetic Analyzer (Rapid_Seq_Assay_XL_POP7). Results were analyzed using SnapGene software (https://www.snapgene.com/, RRID:SCR_015052).

### Cell lysis and western blotting

Five million LCLs treated or not with 500 nM actinomycin D (#BML-GR300; Enzo) for different time points (2–8 h) were harvested and resuspended in lysis buffer (0.1% Triton X-100, 50 mmol/l Tris, pH 7.5, 100 mmol/l NaCl, 0.1 mmol/l EDTA, 10 mmol/l NaF) (#9803; Cell Signaling Technology). Protease/Phosphatase Inhibitor Cocktail (#5872S; Cell Signaling Technology) was added to the lysis buffer immediately before cell lysis. Unstimulated U2OS cells were collected directly from culture flasks, washed once with PBS, and subsequently lysed using the previously described lysis buffer. The cell lysates were incubated for 30 min at 4°C and then centrifuged at 14,000 *g* for 10 min at 4°C. The supernatants were removed, and protein quantities were measured using the Bradford protein assay. Proteins were boiled with Bolt LDS Sample Buffer 4× (B0008; Thermo Fisher Scientific) and β-mercaptoethanol (M3146; Sigma-Aldrich).

Protein extracts (20 μg) from LCLs or U2OS cells were resolved using SDS-PAGE on a NuPAGE 12% Bis-Tris gel (#NP0342BOX; Invitrogen). A Spectra Multicolor Broad Range Protein Ladder (#26634; Thermo Fisher Scientific) was included for molecular weight reference. Proteins were transferred onto a low-fluorescence polyvinylidene difluoride membrane using the iBlot 3 Western Blot Transfer System (Thermo Fisher Scientific). To prevent protein loss from membrane stripping, the membranes were sectioned based on the molecular weight of the target proteins. Each membrane section was blocked with 5% BSA (#GAUBSA01-64; Eurobio) in TBS-T (20×) (#28360; Thermo Fisher Scientific) and incubated overnight at 4°C with gentle agitation with the following primary antibodies: anti-p53 mouse (1:1,000, Cat# sc-126; Santa Cruz Biotechnology, RRID:AB_628082), anti-p21 rabbit (1:1,000, Cat# 2946; Cell Signaling Technology, RRID:AB_2260325), anti-Flag mouse (1:4,000, Cat# F1804; Sigma-Aldrich, RRID:AB_262044), anti-actin mouse (1:1,000, Cat# sc-47778; Santa Cruz Biotechnology, RRID:AB_626632), and anti-α tubulin mouse (1:4,000, Cat# T5168; Sigma-Aldrich, RRID:AB_477579). After washing, the membranes were incubated for 1 h with the appropriate HRP-conjugated secondary antibody (anti-mouse-HRP, Cat# 31430; Thermo Fisher Scientific, RRID:AB_228307 or anti-rabbit-HRP, Cat# 31460; Thermo Fisher Scientific, RRID:AB_228341). Chemiluminescence detection was performed using SuperSignal West Femto Maximum Sensitivity Substrate (Thermo Fisher Scientific) on a ChemiDoc XRS system (Bio-Rad). The resulting images were analyzed using Image Lab 4.0 software (Bio-Rad).

### Immunofluorescence

30,000 cells per condition were plated onto sterile 12-mm glass coverslips and incubated overnight. The following day, the cells were washed once with PBS and fixed with 4% paraformaldehyde for 15 min. After fixation, the cells were rinsed with PBS and permeabilized using 0.5% Triton X-100 in PBS for 10 min. To block nonspecific binding, the cells were incubated for 40 min in a blocking solution consisting of 5% BSA (BSA Fraction V, #GAUBSA01-62; Sigma-Aldrich) in PBS, which had been previously filtered. Primary antibodies: anti-fibrillarin rabbit (1:200, Cat# ab5821; Abcam, RRID:AB_2105785), anti-UBF (1:100, Cat# sc-13125; Santa Cruz Biotechnology, RRID:AB_671403), and anti-Flag either rabbit (1:500, Cat# F7425; Sigma-Aldrich, RRID:AB_439687) or mouse (1:500, Cat# F1804; Sigma-Aldrich, RRID:AB_262044) diluted in PBS with 5% BSA, were incubated overnight at 4°C. Following two washes with PBS, secondary antibodies were added, tailored to the isotype or species of the primary antibody: goat anti-mouse IgG secondary antibody, Alexa Fluor 555 (1:1,000, Cat# A-21422; Thermo Fisher Scientific, RRID:AB_2535844), donkey anti-rabbit IgG, Alexa Fluor 488 (1:1,000, Cat# A-21206; Thermo Fisher Scientific, RRID:AB_2535792); donkey anti-rabbit Alexa Fluor 555 (1:1,200, Cat# A-31572; Thermo Fisher Scientific, RRID:AB_162543), and goat anti-mouse green Alexa Fluor 488 (1:2,000, Cat# A-11001; Thermo Fisher Scientific, RRID:AB_2534069) were incubated for 30 min at room temperature. After three final PBS washes with gentle agitation, the coverslips were mounted using ProLong Gold Antifade Mountant with DNA Stain DAPI (#P36935; Invitrogen). Imaging was performed using a Leica SP8 confocal microscope or a Zeiss spinning disk system (Plateforme d’Imagerie Cellulaire, SFR Necker), and the images were analyzed using Fiji software (version 2.14.0/1.54f, RRID:SCR_002285).

### Generation of WDR75 endogenous clones

Guide RNA for SpCas9 targeting exon 3 of WRD75 was designed using CRISPOR (RRID:SCR_015935). Plasmid pU6-(BbsI)_CBh-Cas9-T2A-mCherry, a gift from Ralf Kuehn (Berlin Institute of Health, Berlin, Germany) (RRID:Addgene_64324), was digested with BbSI restriction enzyme. Two oligos 5′-CAC​CGA​GCT​TGG​GCA​AGA​GTA​AAG-3′ and 5′-AAA​CCT​TTA​CTC​TTG​CCC​AAG​CTC-3′ containing the guide sequence were annealed and cloned into digested pU6-(BbsI)_CBh-Cas9-T2A-mCherry. U2OS cells were transfected with the previous plasmid (Amaxa kit L from Lonza) and sorted 2 days after based on live cells, mCherry+ signal in 96-well plates. Clones were amplified and screened for indel events. The clones were analyzed using one set of primers (5′-CTG​CCT​TGA​TGG​ATC​TTA​TG-3′ and 5′-AAT​AAT​CCC​TCC​CTC​ACT​GC-3′) around the Cas9 cut site. KO score for each sequence was evaluated using Inference of CRISPR Edits (RRID:SCR_024508) software (Performance Analysis, 2019. v3.0; Synthego). Selected “KO clones” were used to extract RNA; cDNA was generated as described above. A PCR was performed to amplify the modified region (5′-CGG​CAG​CGA​GTT​GAA​CTT​TA-3′ and 5′-GGT​TGT​TGG​GGT​TAA​GCT​GG-3′). PCR products were then cloned into TOPO TA vector (#45-0030; Thermo Fisher Scientific) and processed according to the manufacturer’s instructions. At least 8 independent clones per sample were picked. Wizard Plus SV Minipreps DNA Purification System (#A1460; Promega) was used to extract the plasmid DNA from each clone. For the following sequencing BigDye reaction, M13_F 5′-GTA​AAA​CGA​CGG​CCA​GT-3′ was used as a primer.

### Cell proliferation

CellTrace Violet (CTV) was freshly prepared according to the manufacturer’s instructions (Molecular Probes, C34571; Thermo Fisher Scientific) to create a 5-mM stock solution. The stock was diluted 1:2,000 in PBS to prepare a 2.5 μM working solution prewarmed at 37°C for 5 min. T cells were resuspended at a concentration of 1 × 10^6^ cells/ml and washed three times with 1× PBS. Subsequently, the cells were incubated with the diluted CTV solution at 37°C in a darkened water bath for 15 min. Following incubation, the CTV-stained cells were washed twice with 9 ml of RPMI supplemented with SAB and then resuspended in 10 ml of fresh RPMI SAB supplemented with IL-2. The cells were stimulated with anti-CD3/α-CD28 beads at a 1:1 ratio and incubated for 4 days. Finally, the samples were analyzed by flow cytometry. B cells were stimulated with IL-4, IL-21, and CpG for 4 days before analysis.

### Isolation of total RNAs and analysis by northern blot

For total RNA isolation, 5–10 × 10^6^ cells were collected by centrifugation at 250 *g*, and grown in fresh medium for 4 h. They were then rinsed with cold PBS, and the cell pellets were lysed in 1 ml Tri reagent (Molecular Research Center, Inc.). After a first extraction with chloroform according to the manufacturer’s instructions, the aqueous phase was extracted twice with phenol–chloroform–isoamyl alcohol (25:24:1; Sigma-Aldrich), then with chloroform. Total RNAs were recovered after precipitation with 2-propanol. For northern blot analyses, RNAs were separated on a 1.1% agarose gel containing 1.2% formaldehyde and 30 mM triethanolamine, 30 mM tricine, pH 7.9 (3 μg total RNAs/lane), then transferred to a Hybond-N^+^ nylon membrane (GE Healthcare) by passive transfer. In order to analyze 5S and 5.8S rRNAs, 2 µg total RNAs were separated on 6% polyacrylamide gels (19:1) (Bio-Rad) in Tris/borate/EDTA buffer containing 7 M urea, and then electrotransferred to a nylon membrane overnight. After cross-linking under UV light, prehybridization was performed for 2–3 h at 45°C in 6× saline-sodium citrate buffer (SSC), 5× Denhardt’s solution, 0.5% SDS, 0.9 μg/ml tRNA. The 5′-radiolabeled oligonucleotide probe was incubated overnight. After washing twice for 10 min in 2× SSC, 0.1% SDS, and once in 0.5× SSC, 0.1% SDS, the membrane was exposed to a PhosphorImager screen. Radioactive signals were revealed using a Typhoon IP PhosphorImager and quantified using ImageQuant TL software (Amersham). The membranes were then dehybridized in 0.5% SDS, and then rehybridized with another radiolabeled probe. The DNA probes were as follows: 5′ETS_297_: 5′-AGA​CGA​GAA​CGC​CTG​ACA​CGC​ACG​GCA​C-3′; 5′ETS_1399_: 5′-CGC​TAG​AGA​AGG​CTT​TTC-3′; 5′ETS_3636_: 5′-GTA​AGG​TAG​AGC​GCG-3′; 5′ITS1: 5′-CCT​CGC​CCT​CCG​GGC​TCC​GTT​AAT​GAT​C-3′; ITS1-5.8S: 5′-CTA​AGA​GTC​GTA​CGA​GGT​CG-3′; ITS2: 5′-CTG​CGA​GGG​AAC​CCC​CAG​CCG​CGC​A-3′ and 5′-GCG​CGA​CGG​CGG​ACG​ACA​CCG​CGG​CGT​C-3′; 28S-3′ETS: 5′-GCA​CGC​GCG​CGC​GGA​CAA​ACC​CTT​G-3′; 5S: 5′-TTC​CGA​GAT​CAG​ACG​AGA​TCG​GG-3′; 5.8S: 5′-GTT​CTT​CAT​CGA​CGC​ACG​AGC-3′; 18S: 5′-TTT​ACT​TCC​TCT​AGA​TAG​TCA​AGT​TCG​ACC-3′; 28S: 5′-CCC​GTT​CCC​TTG​GCT​GTG​GTT​TCG​CTA​GAT​A-3′; 7SK: 5′-CAT​GGA​GCG​GTG​AGG​GAG​GA-3′ and 5′-GTG​TCT​GGA​GTC​TTG​GAA​GC-3′.

### Polysome profiling

30 × 10^6^ cells per LCL were treated with 50 µg/ml of CHX (Sigma-Aldrich) for 10 min at 37°C. After treatment, the cells were pelleted by centrifugation, the supernatant was discarded, and the pellet was resuspended in 2 ml of 1× hypotonic buffer, supplemented with 0.5% Triton X-100, 0.5% sodium deoxycholate, 120 U/ml RNase inhibitor, 3 mM DTT, and 50 µg/ml CHX. The suspension was centrifuged at 6,000 rpm (3,000 × *g*) for 10 min, and the supernatant was collected. Heparin was added to the supernatant to a final 1 mg/ml concentration from a 50 mg/ml stock solution. Sucrose gradients were prepared using 10% and 50% sucrose solutions, with sucrose dissolved in polysomal buffer containing 15 mM Tris-HCl (pH 7.4), 15 mM MgCl_2_, and 300 mM NaCl, ensuring RNase-free conditions. The clarified supernatants from the lysed cells were layered onto the 10–50% sucrose gradients and centrifuged at 150,000 × *g* for 140 min at 4°C using an SW41 rotor (HITACHI HIMAC CP80WX; Beckman Coulter). After centrifugation, sucrose gradient fractions were collected using ISCO Gradient Fractionation System (ISCO Model 160 Gradient Former and Foxy Jr. Fraction Collector). The absorbance at 254 nm was monitored to record the polysome profile.

### OPP protein staining

LCLs (1 × 10^5^ cells per well) were seeded in a 96-well plate and incubated overnight. The cells were treated with 100 µg/ml CHX for 2 h, after which the medium was removed, the plate was centrifuged for 5 min at 1,500 × *g*, and 100 μl of 20 µM Click-iT OPP working solution was added to each well. The cells were incubated at 37°C for 30 min, and the medium containing Click-iT OPP was then removed. After a PBS wash, cells were fixed with 3.7% formaldehyde in PBS for 15 min at room temperature, followed by permeabilization with 0.1% Triton X-100 in PBS for 10 min. After removing the permeabilization buffer and washing the cells twice with PBS, 100 μl of Click-iT Plus OPP reaction cocktail (prepared with Click-iT OPP reaction buffer 1×, Click-iT reaction buffer additive 1×, 100 mM CuSO_4_ solution, and 10 mM Alexa Fluor 647 azide) was added to each well. The cells were incubated at room temperature for 30 min in the dark, washed once with PBS, resuspended in fresh PBS, and analyzed by flow cytometry to assess Alexa Fluor 647 fluorescence and OPP incorporation.

### Artificial intelligence

Microsoft 365 Copilot was occasionally used to polish, condense, and edit the writing of the manuscript.

### Data analysis

Graphs were plotted using GraphPad Prism v10.4.1. Results are presented as means ± SD. Unless otherwise noted, statistical analysis was performed using the nonparametric Mann–Whitney *U* test. For pre-rRNA precursor quantification in U2OS cells with inactivated *WDR75* gene and ectopic expression of WDR75 WT or WDR75 p.T46I, statistical analysis was performed using paired Student’s *t* test. Western blot quantification was analyzed using one-way ANOVA with multiple comparisons. A P value <0.05 was considered significant.

### Online supplemental material

Fig. S1 shows B cell and T cell proliferation. Fig. S2 presents the 3D structure of the WDR75 protein. Fig. S3 displays data on protein synthesis and polysome profile. Fig. S4 shows that the WDR75 p.T46I variant colocalizes with UBF and fibrillarin, and Fig. S5 shows the inactivation of the *WDR75* gene locus in U2OS cells ectopically expressing WDR75.

## Supplementary Material

SourceData F2 is the source file for Fig. 2.

SourceData F3 is the source file for Fig. 3.

SourceData F4 is the source file for Fig. 4.

SourceData FS5 is the source file for Fig. S5.

## Data Availability

The data supporting the conclusion of this study, including the numerical values underlying the graphical data in Figures, the means reported in the main text and supplemental material, are available from the corresponding author upon reasonable request. Deidentified data from human participants can be provided where permitted. Variant data have been submitted to ClinVar, accession numbers SCV007518010 and SCV007518011.
