# Supplementary figures and images for: Ribosomal RNA processing impairments in a B cell immunodeficient patient with WDR75 variants
Source: J Hum Immun. 2026 May 6;2(4):e20250061. doi: 10.70962/jhi.20250061 (PMC13148477; doi:10.70962/jhi.20250061)

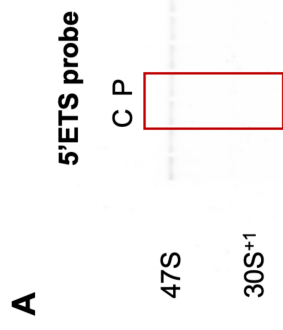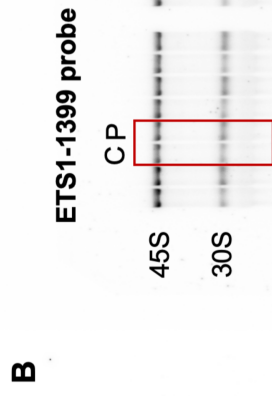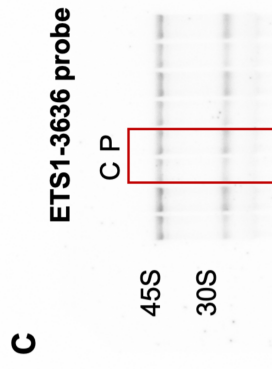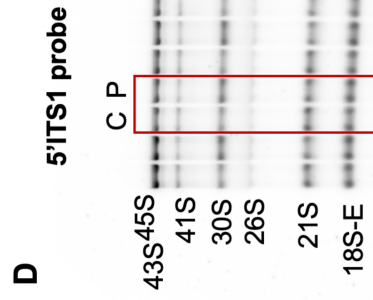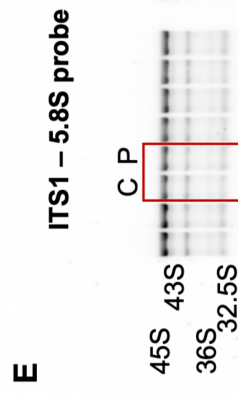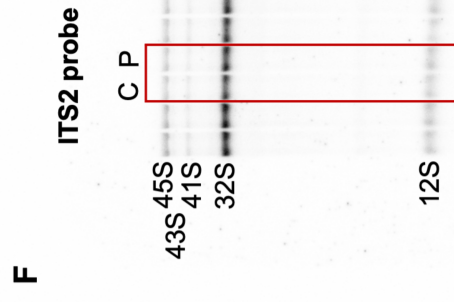

Supplement: SourceData F2 — is the source file for Fig. 2. [file jhi_20250061_sourcedataf2.pdf]

stripping

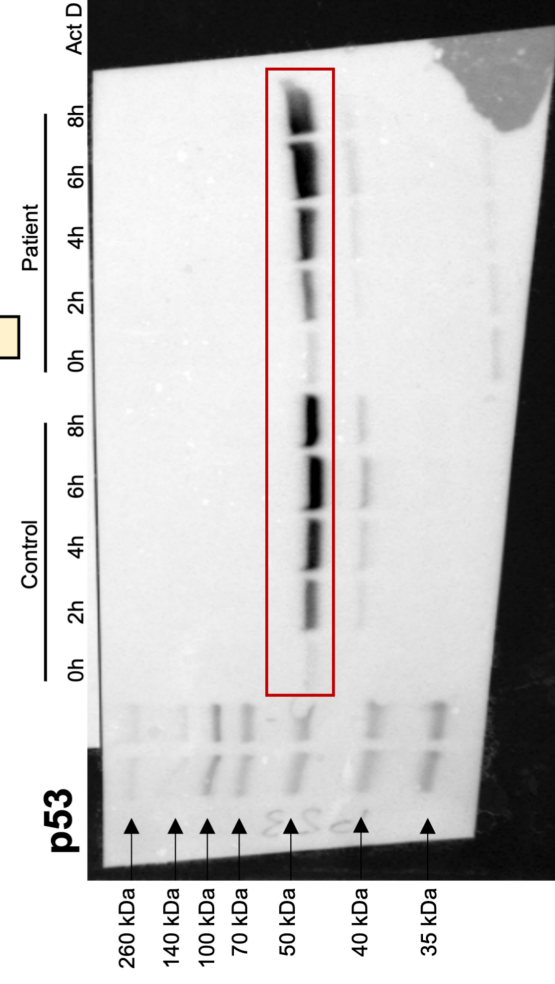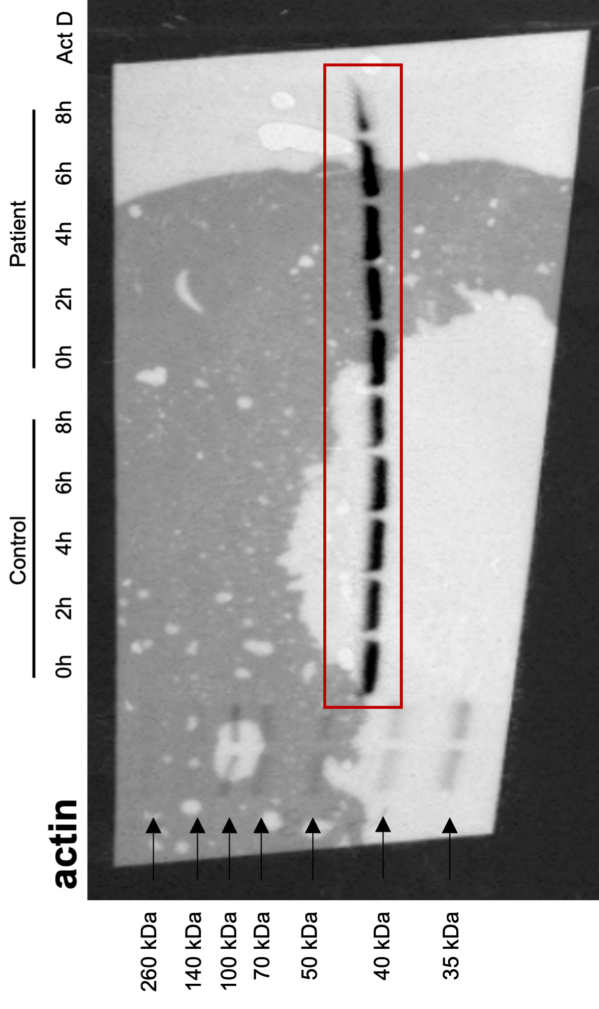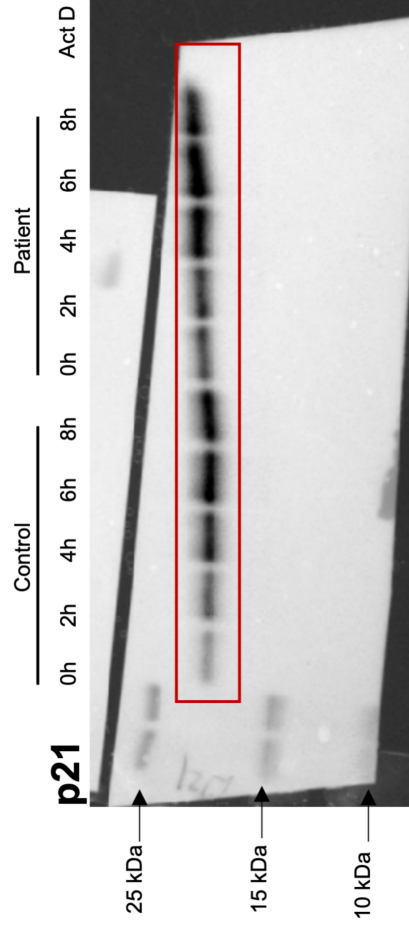

Supplement: SourceData F3 — is the source file for Fig. 3. [file jhi_20250061_sourcedataf3.pdf]
