# Supplementary material for: Ribosomal RNA processing impairments in a B cell immunodeficient patient with WDR75 variants
Source: J Hum Immun. 2026 May 6;2(4):e20250061. doi: 10.70962/jhi.20250061 (PMC13148477; doi:10.70962/jhi.20250061)
Supplement: SourceData F4 — is the source file for Fig. 4. [file jhi_20250061_sourcedataf4.pdf]

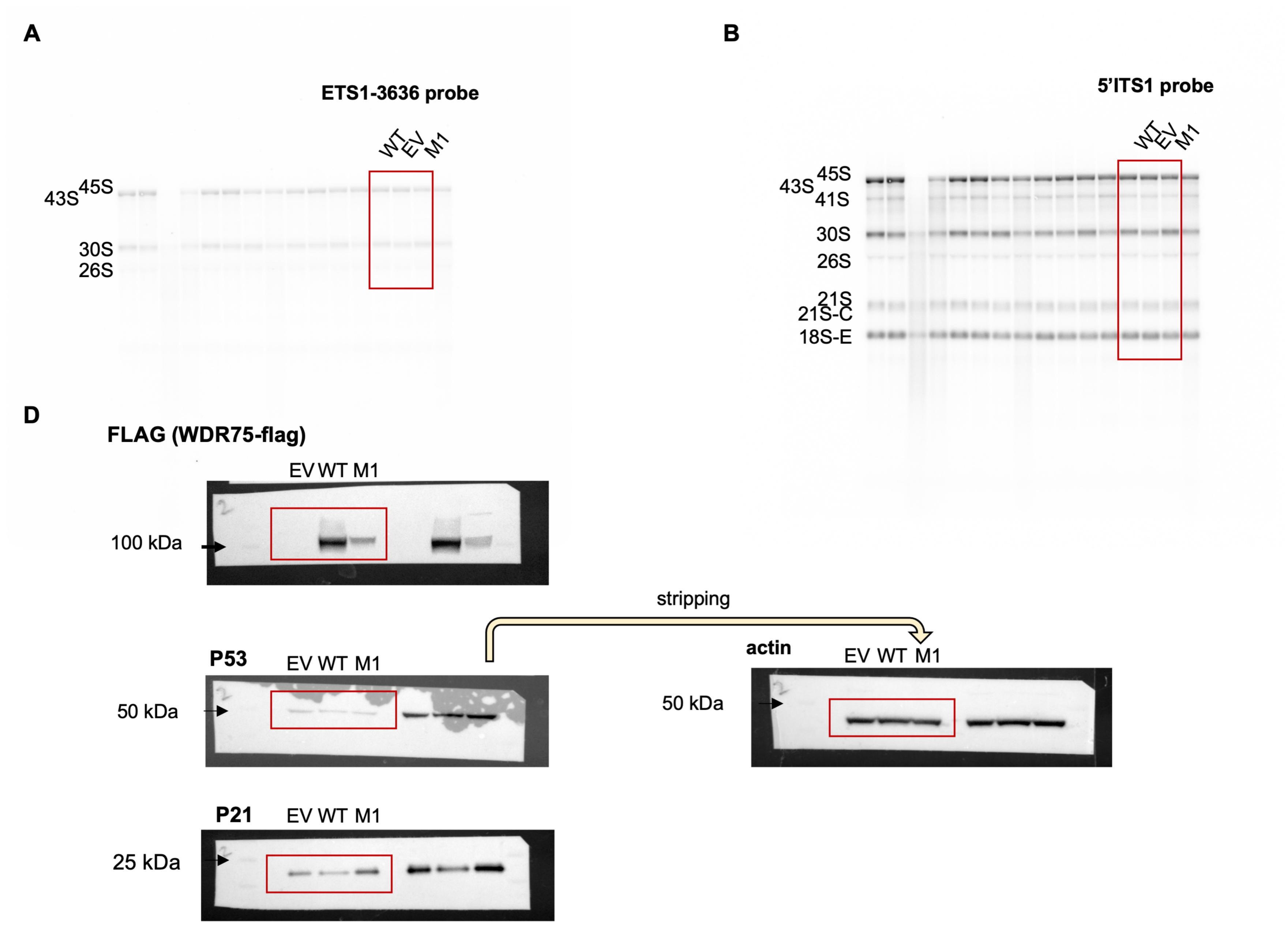
A
ETS1-3636 probe
WT EV M1
45S
43S
30S
26S
B
5'ITS1 probe
WT EV M1
45S
43S
41S
30S
26S
21S
21S-C
18S-E
D
FLAG (WDR75-flag)
EV WT M1
100 kDa
P53
EV WT M1
50 kDa
stripping
actin
EV WT M1
50 kDa
P21
EV WT M1
25 kDa
