# Supplementary material for: Ribosomal RNA processing impairments in a B cell immunodeficient patient with WDR75 variants
Source: J Hum Immun. 2026 May 6;2(4):e20250061. doi: 10.70962/jhi.20250061 (PMC13148477; doi:10.70962/jhi.20250061)
Supplement: SourceData FS5 — is the source file for Fig. S5. [file jhi_20250061_sourcedatafs5.pdf]

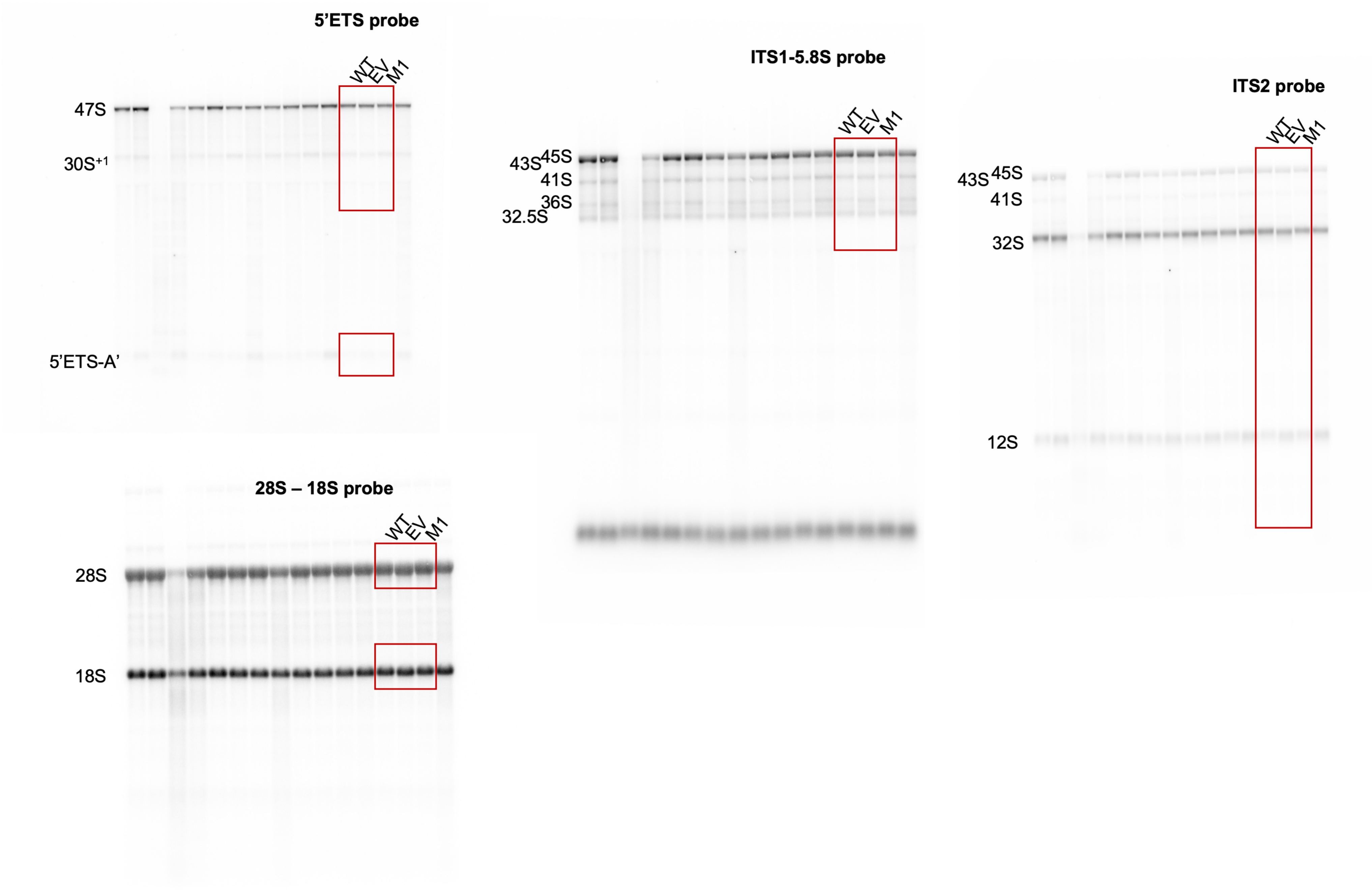
5'ETS probe
WT
EV
M1
47S
30S+1
5'ETS-A'
ITS1-5.8S probe
WT
EV
M1
43S
45S
41S
36S
32.5S
ITS2 probe
WT
EV
M1
43S
45S
41S
32S
12S
28S – 18S probe
WT
EV
M1
28S
18S
